# Efficacy and safety of acupuncture combined with auricular acupressure for smoking cessation: A study protocol of a multicentre, randomized, controlled clinical trial

**DOI:** 10.3389/fneur.2022.921054

**Published:** 2022-07-27

**Authors:** Jinchun Zeng, Yizu Liao, Xiaojing Wei, Guangxian Chen, Zibin Cai, Min Chen, Yanhua Gou, Guohua Lin

**Affiliations:** ^1^Department of Rehabilitation, The First Affiliated Hospital, Guangzhou University of Chinese Medicine, Guangzhou, China; ^2^The First Clinical Medical College, Guangzhou University of Chinese Medicine, Guangzhou, China; ^3^Clinical Research and Data Center, South China Research Center for Acupuncture and Moxibustion, Medical College of Acu-Moxi and Rehabilitation, Guangzhou University of Chinese Medicine, Guangzhou, China; ^4^Department of Chinese Medicine Services, Pok Oi Hospital, Hong Kong, China; ^5^Department of Acupuncture and Moxibustion, Shenzhen Traditional Chinese Medicine Hospital, Shenzhen, China; ^6^Shenzhen Nanshan Hospital of Traditional Chinese Medicine, Shenzhen, China

**Keywords:** acupuncture combined with auricular acupressure (A&AA), smoking cessation, nicotine dependence, acupuncture therapy, auricular acupressure therapy

## Abstract

**Background:**

Nicotine dependence is an addictive behavioral disease facilitated by habitually smoking cigarettes. In many countries, acupuncture and auricular acupressure have attracted growing attention as complementary or alternative treatments for smoking cessation; however, there is a lack of rigorous randomized, controlled studies evaluating the combination of these two interventions specifically for smoking cessation. The aim of this study is to evaluate the efficacy and safety of using acupuncture combined with auricular acupressure (A&AA) to increase the rates of smoking cessation and ultimately reduce the rates of relapse.

**Methods:**

This is a multicentre, prospective, parallel, randomized, controlled trial. A total of 360 patients with severe nicotine dependence will be randomized into test (A&AA) or control (nicotine replacement therapy, NRT) groups. The test group will be treated with A&AA twice weekly, while the control group will use an NRT patch daily. All treatments will be administered for 8 weeks, with a follow-up period of 4 months. The primary outcome will be the smoking abstinence rate at week 24, with a combined safety assessment. The secondary outcomes will be smoking cessation rates at other timepoints, saliva cortisone test results, and scores on the Fagerstrom Test for Nicotine Dependence, the Autonomy over Tobacco Scale, the Hamilton Anxiety Rating Scale, the Self-rating Anxiety Scale, and the Pittsburgh Sleep Quality Index. The cost of treatment will also be used to evaluate the economic effects of different smoking cessation interventions. Statistical analysis on the data collected from both the intention-to-treat (all randomly assigned patients) and per-protocol (patients who complete the trial without any protocol deviations) patients, will be performed using the statistical software package, IBM SPSS 27.0.

**Discussion:**

This study will provide rigorous clinical evidence evaluating the efficacy and safety of using A&AA as a smoking cessation therapy.

**Trial registration:**

Chinese Clinical Trial Registry (Registration number: ChiCTR1900028371).

## Background

Nicotine dependence has been classified as a mental and behavioral disorder by the 2010 International Classification of Diseases codes issued by the World Health Organization. According to research published in the fields of tobacco economics and control, tobacco kills approximately six million people, costs the world's economies more than one trillion dollars annually, and remains one of the major causes of premature death (1). China has the highest rates of cigarette addiction worldwide, especially among male smokers. In China, more than one million people lose their lives each year from smoking-related diseases (2). Diseases and mortality associated with smoking pose a serious threat to public health. The high smoking rate and the difficulty in controlling this addiction suggests that key policymakers should devote significant resources to this issue. In fact, health authorities in many countries have strongly recommended that doctors intervene to help patients quit smoking.

The main methods for achieving smoking cessation are nicotine replacement therapy (NRT), antidepressant medications, and psychological counseling, with various studies confirming that these methods improve the success rate of smoking cessation to varying degrees (3, 4). Unfortunately, these therapies also have potential side effects, including chest tightness, insomnia, dry mouth, skin allergies, and gastrointestinal reactions (5–12), which can reduce patient compliance and most importantly, the efficacy of smoking cessation.

Since 2015, complementary and alternative medicine for substance use disorders has increasingly gained attention, and acupuncture may be a hotspot in this field (13). Various types of acupuncture, such as hand acupuncture, ear acupuncture, laser acupuncture, have gained attention in many countries as therapeutic interventions for smoking cessation (14). In a smoking cessation trial in Norway (15), the experimental group received an acupuncture intervention at the “Shenmen,” “Mouth,” and “Liver” acupoints of the ear, as well as at the acupuncture points, Kongzui (LU 6) and Lieque (LU 7), leading to significant changes in the taste of cigarettes and the desire to smoke, compared with the control group. A multicentre, randomized trial of 300 patients in China (16) also showed that the effects of acupuncture on smoking cessation were not inferior to NRT. Available evidence supports the acupuncture therapy have very wide popularization and application prospects in smoking cessation, while it is also suggested that single type acupuncture therapy is relatively insufficient in the long term of the abstinence rate, and the evidence is insufficient in the efficacy of combined acupuncture therapy (17–19). Thus, we carry out this RCT to observe the effect of combined acupuncture therapy on smoking cessation.

This multicentre, randomized, controlled clinical trial, which is based on a previous study of acupuncture for smoking cessation, will be the first to evaluate whether the combination of a short-term but strong stimulation, elicited by acupuncture and the long-term, milder stimulation elicited by auricular acupressure, will lead to better smoking cessation outcomes. Additionally, we hope to determine whether this treatment (A&AA) has better smoking cessation outcomes and is safer than NRT. We also hope to evaluate the benefits of A&AA from the perspective of health economics, in order to develop effective and affordable smoking cessation plans.

## Methods

### Study design

**Figure 1:**
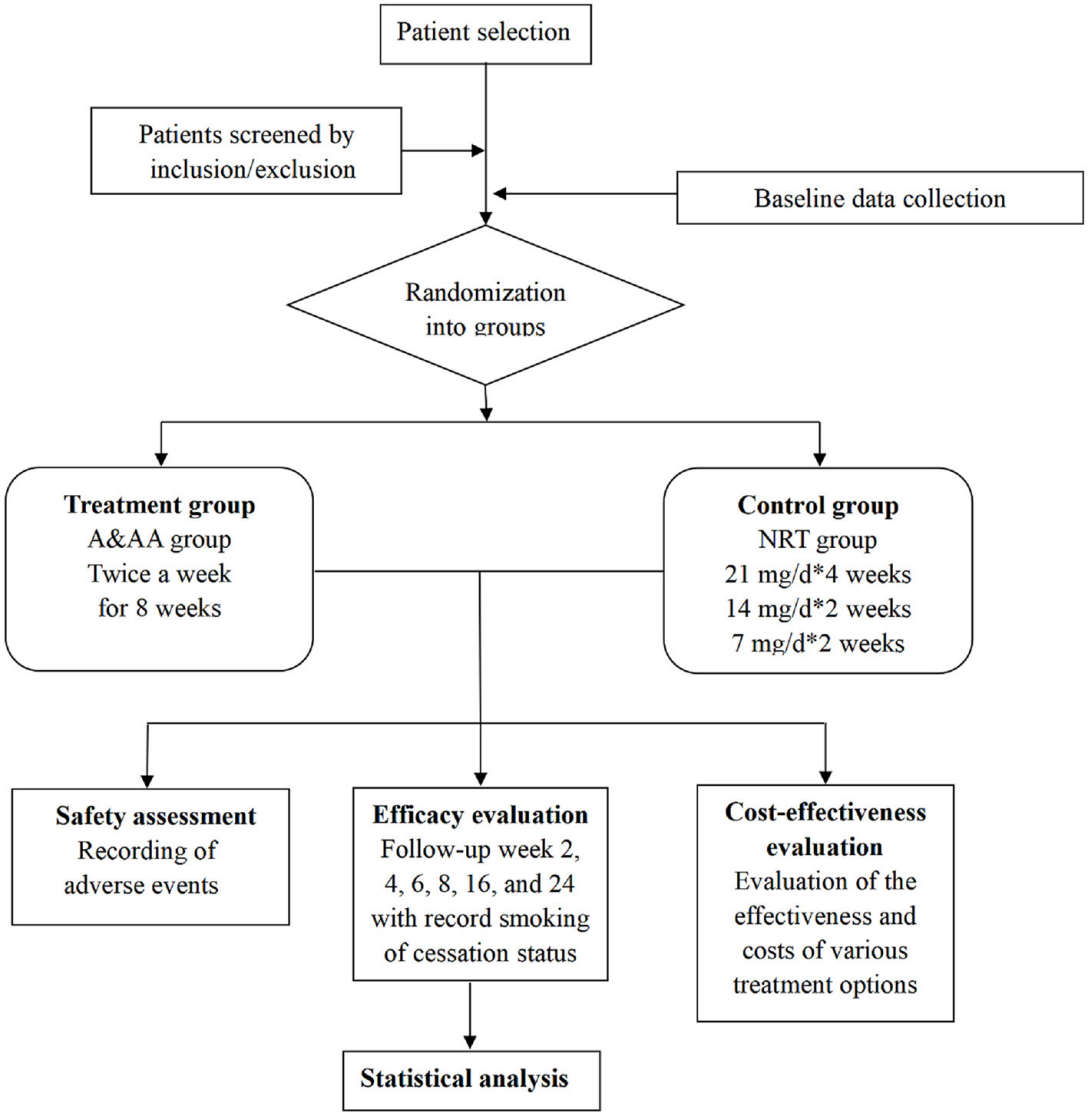
Patient selection and methodological flow.

For this study, a multicentre, prospective, randomized controlled trial will be conducted to compare the effects of A&AA with those of NRT, on smoking cessation. A total of 360 patients with severe nicotine dependence, who are willing to voluntarily quit smoking, will be randomly divided into a treatment group (A&AA) or a control group (NRT). The First Affiliated Hospital of Guangzhou University of Chinese Medicine, the Hong Kong Pok Oi Hospital, and the Shenzhen Chinese Medicine Hospital will be responsible for patient recruitment, screening, interventions, and the follow-up of 280, 30, and 50 patients, respectively. All outcomes will be assessed at The First Affiliated Hospital of Guangzhou University of Chinese Medicine. Management of the randomization sequence, blinding, and data analyses, will be carried out at the Clinical Research and Data Center of Guangzhou University of Chinese Medicine. Ethical approval for this study was received from the First Affiliated Hospital of Guangzhou University of Chinese Medicine (No. ZYYECK [2019] 099), and this study will be conducted according to the Declaration of Helsinki, 7th revision (2013). Informed written consent will be obtained from all patients. The flow diagram for patient selection and methodology is shown in Figure 1. This protocol is reported in accordance with the Standard for Reporting Interventions in Clinical Trials of Acupuncture recommendations (STRICTA) (20).

### Patients

Smokers have the intention to quit smoking and meet our following criteria will be recruited at three sites simultaneously. Posters, leaflets and WeChat advertisements will be used in the recruitment process. Formal enrollment is only considered if participants signed written informed consent.

### Inclusion criteria

Patients who meet all the following criteria will be included: (1) Meet the diagnosis of ICD-10 Nicotine Dependence; (2) between 18 and 65 years of age; (3) smoking history of ≥1 year; (4) currently smokes ≥20 cigarettes per day over the past 1 year; (5) detection of cotinine in saliva; (6) a Fagerstrom Test for Nicotine Dependence (FTND) score of ≥4; (7) willingness and ability to sign a consent form, ensuring that they understand the trial and will take part in it voluntarily; and (8) an elution period of more than 1 month for all smoking cessation therapies, including acupuncture, auricular acupressure, and NRT.

### Exclusion criteria

Patients will be ineligible to participate in this trial if they meet any of the following criteria: (1) severe heart, lung, brain, or blood system diseases or diabetes; (2) drug use or those diagnosed with mental illnesses; (3) history of stroke or nervous system diseases; (4) unexplained symptoms; (5) history of a coagulation disorder or anticoagulant drug history; (6) moderate or severe impairment of liver or kidney function; (7) pregnant or breast feeding; or (8) already using smoking cessation therapies, such as acupuncture, auricular acupressure, NRT, etc.

### Elimination criteria

Patients will be eliminated from the trial based on the following criteria: (1) patients who are willing to participate, but fail to meet the inclusion criteria, and (2) those who fail to complete instructions or procedures with obvious implications for efficacy or safety.

### Termination criteria

The trial will be terminated if the following situations occur during its course: (1) therapies exacerbate the state of the illness or lead to secondary infections; (2) external factors worsen the patients' conditions; or (3) patients are unwilling to continue the trial.

### Sample size

According to a previous randomized, controlled study of acupuncture for smoking cessation (15), we expect that the success rates for smoking cessation will be on average 44% in the NRT (control) group and 60% in the A&AA (treatment) group, using the abstinence rate for smoking cessation at week 24 (24-h carbon monoxide [CO] clearance rate of <10 parts per million) as the primary outcome. Using PASS 2008 software, the sample size required for each group was estimated to be 152 cases by two independent sample rate comparison methods.

Therefore, considering an expected loss of 15% during the follow-up period, we will recruit a total of 360 (≈304/0.85) volunteers for this study. Allocation into the two groups will be assigned in a 1:1 ratio, and each group size will be set at 180.

### Randomization and allocation

Eligible patients will be randomly assigned to receive A&AA or NRT *via* a central randomization system (Clinical Research and Data Center, Guangzhou University of Chinese Medicine). Random numbers will be generated using the stratified block method by a designer from the Clinical Research and Data Center, not involved in the study. Because of the obvious differences between the two types of therapies, blinding of patients and healers will not be possible; therefore, only outcome evaluators will be blinded to the treatment allocation.

### Interventions

Patients with chronic diseases who are taking medication can continue to take medication as prescribed by specialist doctors during the phase of this trial.

### A&AA group

Patients in this group will receive acupuncture and ear acupuncture treatment in sequence delivered by licensed acupuncturist with at least 5 years of therapeutic experience. Locations of all the acupoints are in line with the National Standard of the People's Republic of China's (GB/T 12346–2006) standard.

**Figure 2:**
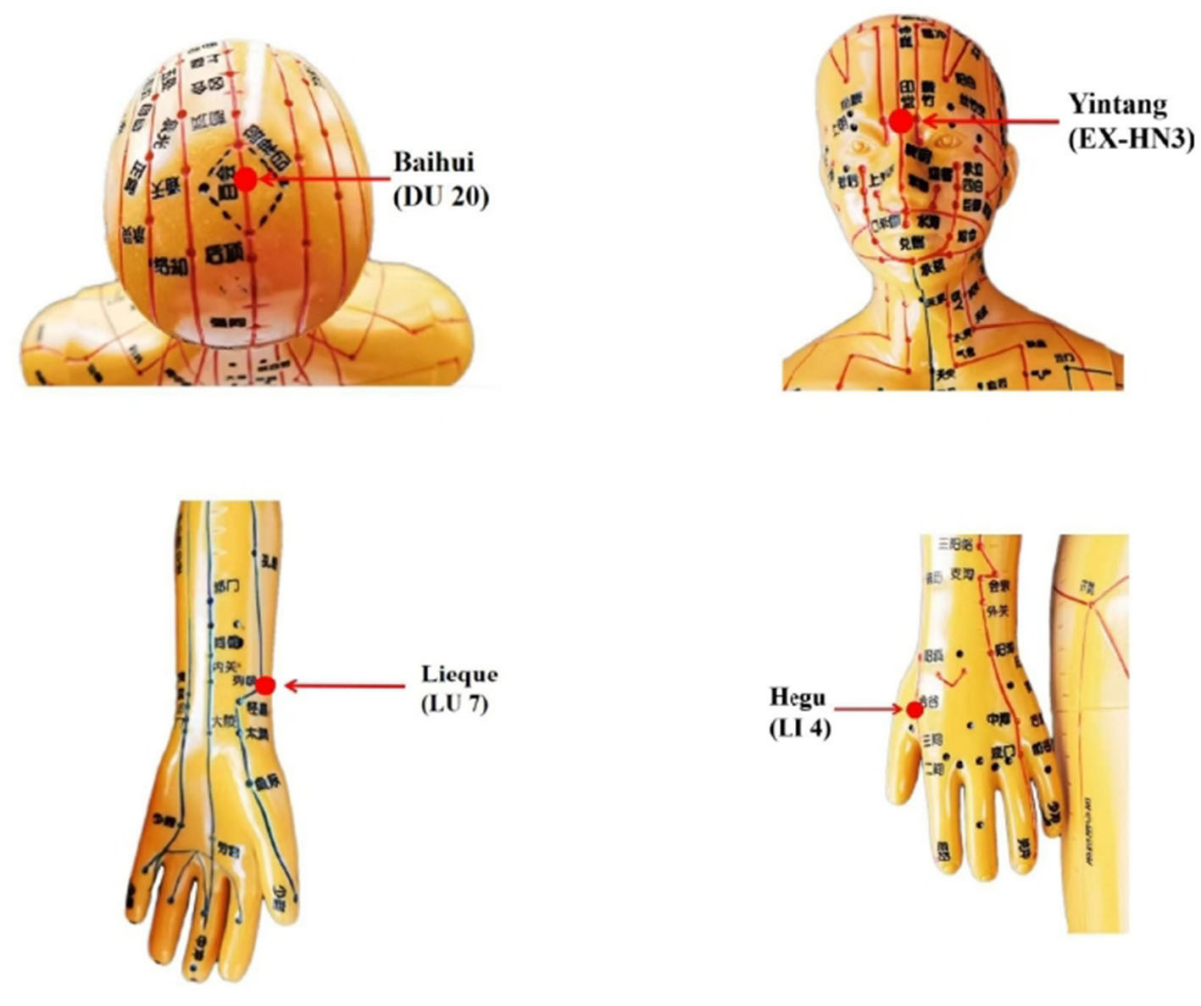
Acupuncture points.

Manual acupuncture acupoint prescriptions include:Baihui (DU 20), Yintang (EX-HN3), bilateral Lieque (LU 7), and bilateral Hegu (LI 4) (Figure 2). The acupuncture procedure will be as follows: Before acupuncture, patients will be placed in a supine position to expose the acupoints. After skin disinfection, 40-mm disposable sterile needles will be inserted horizontally at Baihui (GV 20), bilateral Hegu (LI 4), and bilateral Lieque (LU 7, point to Yangxi), and a 25 mm needle will be inserted vertically at Yintang (EX-HN3). The neutral supplementation and draining method will be applied at all acupoints for 30 min after achieving the arrival of qi; the needle will be manipulated every 10 min to avoid discomfort (needle sensation as soreness or numbness).

**Figure 3:**
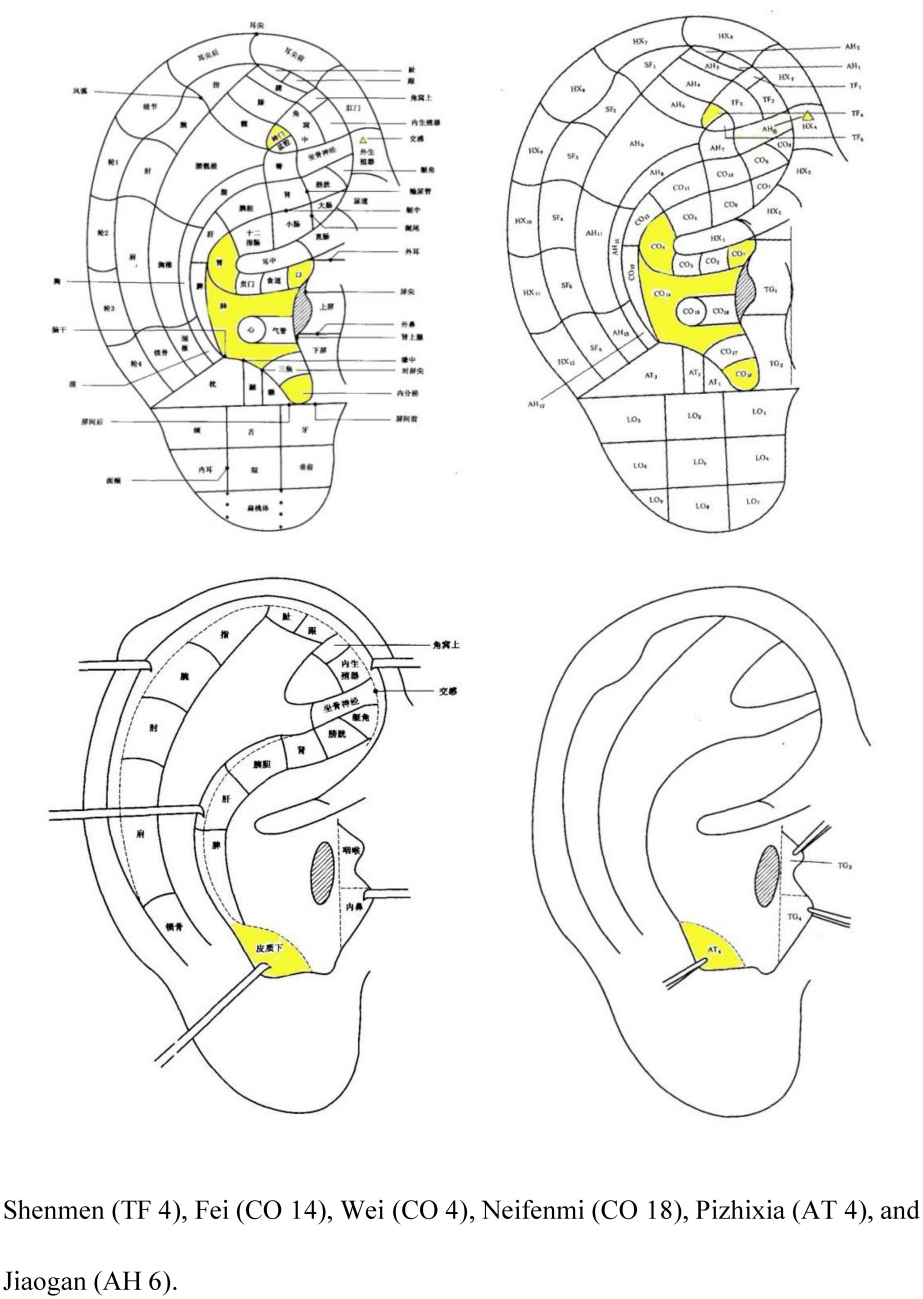
Nomenclature and location of auricular points (21).

After the acupuncture procedure, auricular compression at Shenmen (TF 4), Fei (CO 14), Wei (CO 4), Neifenmi (CO 18), Pizhixia (AT 4), and Jiaogan (AH 6) (Figure 3) will be applied. The acupuncture procedure will be as follows: after routine disinfection of the auricle and ear circumference, a patch of vaccaria seeds will be stuck to the auricular acupoints mentioned above on one ear (contralateral to the ear on which this technique was performed 3-day earlier). In addition, patients will be instructed to press each auricular acupuncture point for 60 s, 3–5 times per day.

The A&AA interventions will be performed simultaneously twice a week for a total treatment course of 8 weeks.

### NRT group

Patients in the NRT group will receive NRT patches purchased from Novartis (approval number: N07BA01) which are composed of nicotine at three different dosages, including 21, 14, and 7 mg. After removing the protective foil, the patch will be applied to a clean, dry, intact area of skin (free from lotion, alcohol, or ointment), preferably on the trunk or otherwise on the upper arm or hip, and pressed with the palm for 10 s. To avoid local irritation of the skin, a different site of application will be chosen each day. Patients will be asked to use one patch per day and visit the outpatient clinic twice per week. Treatment will start at 21 mg/d for 4 weeks, followed by 14 mg/d for 2 weeks, and 7 mg/d for the final 2 weeks. The total course of treatment will be 8 weeks.

### Strategies to improve adherence

All the therapies and medical examination items in this trial will be provided to the patients free of charge, and patients who can complete all the visits will be reimbursed the transportation expenses. These measures will improve the patients' compliance.

### Outcome measures

#### Primary outcome

The abstinence rate for smoking cessation at week 24 (number of people who quit smoking/total number of people) will be considered the primary outcome. Patients with a 24-h CO clearance rate of <10/1,000,000 at the 24th week of therapy, will be considered to have successfully quit smoking.

#### Secondary outcomes

The secondary outcomes will be the prolonged abstinence rate, the abstinence rate at other time points, the results from a saliva cortisone test, and scores on the Fagerstrom Test for Nicotine Dependence (FTND), the Autonomy over Tobacco Scale (AUTOS), the Hamilton Anxiety Rating Scale (HAM-A), the Self-rating Anxiety Scale (SAS), and the Pittsburgh Sleep Quality Index (PSQI). Additionally, we will conduct safety assessments of and evaluate cost of treatment.

### Detection time point of outcomes

Baseline will be recorded before clinical trial, safety will be conducted at the beginning and end of trial. The clinical symptoms, efficacy observation indicators, laboratory indicators and scale evaluation of the patients were examined before treatment and at the follow-up of 2, 4, 6, 8, 16 and 24 weeks after treatment.

All the above results will be timely and truthfully filled in the clinical case report form (CRF).

### Assessment of adverse events

All adverse events will be reported in detail in an observation table (Table 1), including the type of event, the extent of symptoms or diseases, the date of onset, frequency, duration, the remission date, treatment measures, treatment process, results, and follow-up, for evaluation of any correlations between adverse reactions and A&AA or NRT.

**Table 1:** Description of the study schedule.

**Task**	**Screening**	**Observation period**				**Follow-up period**		**Unplanned follow-up**
	**Visit 0** **Week 0**	**Visit 1** **Week 2**	**Visit 2 Week 4**	**Visit 3** **Week 6**	**Visit 4 Week 8**	**Visit 5** **Week 16**	**Visit 6 Week 24**	
Medical history collection	√	–	–	–	–	–	–	–
Signing informed consent	√	–	–	–	–	–	–	–
Inclusion criteria	√	–	–	–	–	–	–	–
Exclusion criteria	√	–	–	–	–	–	–	–
Basic information	√	–	–	–	–	–	–	–
Vital signs	√	√	√	√	√	√	√	√
Blood and urine tests	√	–	–	–	√	–	√	
Liver and kidney function	√	–	–	–	√	–	√	
Respiratory carbon monoxide determination	√	√	√	√	√	√	√	
Coagulation	√	–	–	–	√	–	√	
Blood lipids + HCY	√	–	–	–	√	–	√	
Saliva cotinine test	√	–	–	–	–	√	√	
ECG	√	–	–	–	–	–	√	
FTND	√	√	√	√	√	√	√	
AUTOS	√	√	√	√	√	√	√	
HAM-A	√	√	√	√	√	√	√	
SAS	√	√	√	√	√	√	√	
PSQI Scale	√	√	√	√	√	√	√	
Adverse event record	–	√	√	√	√	√	√	
Combined medication records	√	√	√	√	√	√	√	
Termination test evaluation	–	√	√	√	√	√	√	

*AUTOS, Autonomy over Tobacco Scale; ECG, electrocardiogram; FTND, Fagerstrom Test for Nicotine Dependence; HAM-A, Hamilton Anxiety Rating Scale; SAS, Self-rating Anxiety Scale; PSQI, Pittsburgh Sleep Quality Index.*

*“√” is recorded when necessary*.

### Assessment of health economics

A cost-effectiveness analysis from a societal perspective will be performed. Cost information, including that regarding medical and non-medical costs, will be collected during treatment, at the end of treatment, and at week 24. The effectiveness index will be considered the primary outcome (Table 1). Cost-effectiveness ratio (CER) and incremental cost-effectiveness ratio (ICER) will be calculated to compare the difference in the cost and clinical outcomes between the two groups. The stability of the results will be tested using sensitivity analysis by the means of bootstrapping and presented with cost-effectiveness acceptability curves (CEAC).

### Data management and monitoring

The data will be monitored by the Electronic Data Capture System (EDC) of the Clinical Research and Data Center of Guangzhou University of Chinese Medicine. Dynamic monitoring of patient enrolment, data entry, and quality verification will be performed by this system in order to strengthen data accuracy and maintain data quality. Study Completed Statistical results will be public access file upload in Chinese Clinical Trial Registry. Participants' personal information will be kept confidential before, during and after the trial.

### Statistical analysis

Data management and statistical analyses for this study will be carried out blingdingly by an independent third party (Clinical Research and Data Center, Guangzhou University of Chinese Medicine). The statistical tests involved in this study will include unilateral and bilateral tests with a significance level of α = 0.05. Statistical analysis will be completed using IBM SPSS 27.0.

All Subjects who will be randomized to undergo at least one treatment comprised the Intention-To-Treat (ITT) population of the study. Subjects who will be randomized into groups and underwent at least one treatment constitute the Safety group of this study. The Per-Protocol analysis data set (PPS), which refers to the cases that comply with the study plan, with the following conditions: the primary variables are clear, no baseline variables are missing, completed all scheduled studies for the 8-week treatment period, and subjects who seriously violate the protocol will be excluded. The safety dataset will be analyzed for subjects who have received at least one treatment after randomization. General demographic, clinical characteristics, and other baseline data will be used to compare the balance between the two groups. Primary outcome measures will be analyzed using ITT population, secondary outcome measures and other outcomes will be analyzed using PPS population. The safety analysis will use the safety population.At each time point, descriptive analyses will be performed to show the two groups of different means (SD and 95% CI) and the rate of outcome change. If the data follow normal distribution, the independent sample *T*-test will be used for comparison. If not, the rank sum test is applied. In order to distinguish therapeutic effects from temporal effects, analysis will be performed using a repeated measurement design.

### Safety analysis

Patients will be questioned about adverse events at each visit, and all adverse events will be recorded and assessed by investigators to identify any causal relationships with the treatment. Blood tests, urinalysis, and electrocardiographic examinations will be performed before and after treatment. Furthermore, Blood lipids, kidney and liver function tests will be re-examined at weeks 16 and 24, and all abnormal changes from the baseline laboratory tests will be evaluated by investigators. Patients will have to report any adverse events that occur at any time to the study team.

Safety will be evaluated by tabulations of adverse events and will be presented with descriptive statistics at baseline and at follow-up visits for each treatment group. All details about adverse events, such as timing, severity, treatment, and causality to the intervention, will be recorded in CRF tables and descriptive statisticswill be performed. Chi-square test or Fisher's exact probability will be used to compare the incidence of adverse events between the two groups, and rank sum test will be used to compare the severity of adverse events.

### Handling of missing data

In the event of a nonresponse after follow-up, the subjects will be informed by our researchers and be asked to provide the missing data. Some loss to follow-up is expected over the 24 weeks. The proportion of patients with missing data for each outcome will be summarized in each group and at each timepoint. If there is <5% of data missing for a specified primary or secondary outcome, we will perform a complete case analysis without imputing the missing values. If there is more than 5% of data missing, we will perform fewer tests. We will continue to analyse all the cases without imputing missing values if the complete case dataset is indicated by fewer tests to be a random sample. If the complete case dataset is not indicated to be a random sample by Little's test, we will then report the point estimates and their 95% confidence intervals by applying the worst- and best-case scenario imputations for the missing values. Multiple imputations will not be performed if the worst- and best-case analyses allow for the same conclusion. Otherwise, multiple imputations will create 10 imputed datasets under the assumption of data missing at random. The results of the trial will be a pooled intervention effect and 95% confidence interval of the analyses of each data set after multiple imputations.

### Quality control

To control quality in multi-centers, we have developed some strategies: firstly, elaborate clinical documents, such as investigator's brochure, case report form, acupuncture operation guideline, are prepared in electronic and paper versions for all the researchers; secondly, pre-trial training is required for all researchers which contains the introduction to the entire study execution, demonstration of standardized acupuncture practice procedures, procedures for follow-up and implementation of the questionnaire, etc.; thirdly, CRFs from various centers will be extracted and checked against data from original medical records; finally, on-site monitor will put into effect regularly by specified managers to find out and fix up the practical issue in the process of trial in time.

## Discussion

At present, most RCTs on acupuncture for smoking cessation usually use blank controls or fake acupuncture for comparison (22). These studies cannot directly and objectively show the effect of acupuncture on smoking cessation. In 2015, the clinical guideline for Smoking cessation in China (23) recommended three drug therapies: NRT, bupropion, and varenicline. To objectively prove if A&AA can aid successful quitting of smoking, it is necessary to compare it with the drug treatments described in the clinical guidelines. In Jang et al.'s clinical study on smoking cessation, the control group received only NRT and counseling, and the trial group received Traditional Chinese Medicine (TCM). The study found that the effect of smoking cessation was improved using TCM, but the increase of TCM treatment cost had no statistical significance on whether TCM could improve the success rate of smoking cessation (24). Our study design is different in that we compared A&AA and NRT to verify if acupuncture is superior to NRT for smoking cessation. Acupuncture may have greater safety, fewer side effects, while being cost-effective. In China, hospitals at all levels set up acupuncture or TCM clinics, and people who want to quit smoking can easily find A&AA intervention. However, psychological counseling and smoking cessation medicine are not widely available, and it is difficult for people to avail these services.

This study will investigate the effectiveness, safety, and economic benefits of the use of A&AA for smoking cessation. This protocol is designed to be a prospective, parallel, multicentre, large-scale, randomized controlled clinical trial. In this trial, Nicotine patches will be provided to the control group as a form of NRT, since all licensed forms of NRT (gum, transdermal patch, nasal spray, inhalator, and sublingual tablets/lozenges) have been reported to help people increase their chances of successfully stopping smoking and to reduce nicotine withdrawal symptoms.

The treatment group will receive A&AA treatment. We designed acupoint combination of acupuncture and ear acupuncture based on traditional acupuncture theory and previous literature studies (22, 25–28). Baihui and Yintang can connect the Du meridian to regulate the consciousness and calm the mind which are reported to reduce dependence, relieve anxiety for addicts (29). Lieque, a acupoint of lung meridian connected with Conception Vessel, can diffuse and regular the lung qi as well as nourish yin-fluid to relieve pharyngeal discomfort in smokers (30). Acupuncture at Hegu can regulate qi movement of spleen and stomach to relieve the discomfort caused by smoking cessation (31). Auricular acupuncture was incorporated into our treatment strategy with the aim of working with manual acupuncture to produce a longer treatment effect for patients to achieve a longer and more stable abstinence rate. Likewise, auricular acupoints play a role in tranquilizing the mind and regulating the Qi of lung and stomach. And it is reported that the continuous stimulation of auricular acupoints will produce the release of neurotransmitters and the change of endocrine that conducive to smoking cessation (32–34).

The limitations of this trial must be acknowledged. First, due to the long experimental and follow-up periods, a high dropout rate may occur, especially in the treatment group. Because of busy professional lives, job mobility, or the lack of acupuncture knowledge, patients may lack the motivation to complete a 24-week course. To reduce this dropout rate, the researchers will have to fully explain the process during the initial stage and will maintain positive contact with volunteers. Second, because this is an unblinded design, there is the possibility of bias due to the expectations or biases of the participants or research staff. To minimize this potential source of bias, researchers will provide public education about acupuncture for smoking cessation, including the principles of acupuncture, and will share stories of successful cases. Last but not least, we must acknowledge that this design lacks a professional psychologist to educated patients, although an educational video has been recorded and all operators have been trained.

So far, the research program has been recruiting patients for 10 months at three centers and patients have already been incorporated into the study. Moreover, 67 patients have decreased their smoking. This preliminary data suggest that A&AA treatment may be promising. This study will provide more evidence to support the use of acupuncture therapy for cessation syndrome, which has been a major concern in recent years.

## Conclusion

In summary, we will apply appropriate clinical trial methods to create a scientific design based on the previous randomized controlled trials on acupuncture use for smoking cessation. We will report the results of this clinical trial in accordance with the international norms of the Consolidated Standards for Reporting of Trials 2010 (CONSORT, 2010). We anticipate that the results of this trial will provide rigorous scientific and clinical evidence to inform smoking cessation programs in severely tobacco-dependent individuals.

## Author contributions

Conception and design: JZ. Administrative support: MC and YG. Provision of study materials or patients: GL, MC, and YG. Collection and assembly of data: YL, ZC, and GC. Data analysis and interpretation: XW. Manuscript writing and final approval of manuscript: All authors.

## Funding

This work was supported by the Guangdong Provincial Administration of Traditional Chinese Medicine Foundation (Nos. 20204005 and 20211128), the Innovative Research Fund of the First Affiliated Hospital of Guangzhou University of Chinese Medicine (No. 2019IIT12), the Guangzhou Science and Technology Project (No. 202102010505), and Sanming Project of Medicine in Shenzhen Nanshan (No. SZSM202103010). Funds are mainly used to purchase materials (such as acupuncture needles, NRT patches, etc.), instruments, and pay labor fees, etc.

## Conflict of interest

The authors declare that the research was conducted in the absence of any commercial or financial relationships that could be construed as a potential conflict of interest. The handling editor CT declared a shared parent affiliation with the authors JZ, YL, XW, GC, ZC, YG, and GL at the time of review.

## Publisher's note

All claims expressed in this article are solely those of the authors and do not necessarily represent those of their affiliated organizations, or those of the publisher, the editors and the reviewers. Any product that may be evaluated in this article, or claim that may be made by its manufacturer, is not guaranteed or endorsed by the publisher.

## Abbreviations

A&AA: acupuncture combined with auricular acupressure
NRT: nicotine replacement therapy
TCM: Traditional Chinese Medicine.

## References

[1] WHO. Tobacco Free Initiative. Available online at: http://www.who.int/tobacco/healthpriority/en/ (accessed September 10, 2018).

[2] Yu B Wang X. Overview of smoking addiction mechanism. Chin J Drug Abuse Prev. (2013) 19:275–8. 10.3969/j.issn.1006-902X.2013.05.009

[3] Zhu S Melcer T Sun J Rosbrook B Pierce JP. Smoking cessation with and without assistance: a population-based analysis. Am J Prev Med. (2000) 18:305–11. 10.1016/S0749-3797(00)00124-0 10788733

[4] Hartmann-Boyce J Chepkin SC Ye W Bullen C Lancaster T. Nicotine replacement therapy versus control for smoking cessation. Cochrane Database Syst Rev. (2018) 5:CD000146. 10.1002/14651858.CD000146.pub5 29852054 PMC6353172

[5] Mills EJ Wu P Lockhart I Wilson K Ebbert JO. Adverse events associated with nicotine replacement therapy (NRT) for smoking cessation. A systematic review and meta-analysis of one hundred and twenty studies involving 177,390 individuals. Tob Induc Dis. (2010) 8:8. 10.1186/1617-9625-8-8 20626883 PMC2917405

[6] Howes S Hartmann-Boyce J Livingstone-Banks J Hong B Lindson N. Antidepressants for smoking cessation. Cochrane Database Syst Rev. (2020) 4:CD000031 10.1002/14651858.CD000031.pub5 PMC7175455 32319681

[7] Han JS Terenius L. Neurochemical basis of acupuncture analgesia. Annu Rev Pharmacol Toxicol. (1982) 22:193–220. 10.1146/annurev.pa.22.040182.001205 7044284

[8] Han JS Ding XZ Fan SG. Cholecystokinin octapeptide (CCK-8): antagonism to electroacupuncture analgesia and a possible role in electroacupuncture tolerance. Pain. (1986) 27:101–15. 10.1016/0304-3959(86)90227-7 3491355

[9] Nutt DJ. Addiction: brain mechanisms and their treatment implications. Lancet. (1996) 347:31–6. 10.1016/S0140-6736(96)91561-5 8531549

[10] Fowler JS Volkow ND Wang GJ Pappas N Logan J MacGregor R . Inhibition of monoamine oxidase B in the brains of smokers. Nature. (1996) 379:733–6. 10.1038/379733a0 8602220

[11] Cabioglu MT Ergene N Tan U. Smoking cessation after acupuncture treatment. Int J Neurosci. (2007) 117:571–8. 10.1080/00207450500535289 17464775

[12] Kang HC Shin KK Kim KK Youn BB. The effects of the acupuncture treatment for smoking cessation in high school student smokers. Yonsei Med J. (2005) 46:206–12. 10.3349/ymj.2005.46.2.206 15861492 PMC2823015

[13] Junyue J Siyu C Xindong W Qinge X Jingchun Z Liming L . Complementary and alternative medicine for substance use disorders: a scientometric analysis and visualization of its use between 2001 and 2020. Front Psychiatry. (2021) 12:722240. 10.3389/fpsyt.2021.722240 34803755 PMC8604152

[14] Wang JH van Haselen R Wang M Yang GL Zhang Z Friedrich ME . Acupuncture for smoking cessation: a systematic review and meta-analysis of 24 randomized controlled trials. Tob Induc Dis. (2019) 17:48. 10.18332/tid/109195 31516491 PMC6662782

[15] He D Berg JE Høstmark AT. Effects of acupuncture on smoking cessation or reduction for motivated smokers. Prev Med. (1997) 26:208–14. 10.1006/pmed.1996.0125 9085389

[16] Wang YY Liu Z Wu Y Yang L Guo LT Zhang HB . Chinese acupuncture for tobacco cessation research team. Efficacy of acupuncture is noninferior to nicotine replacement therapy for tobacco cessation: results of a prospective, randomized, active-controlled open-label. Trial Chest. (2018) 153:680–8. 10.1016/j.chest.2017.11.015 29175360

[17] Dai R Cao Y Zhang H Zhao N Ren D Jiang X . Comparison between acupuncture and nicotine replacement therapies for smoking cessation based on randomized controlled trials: a systematic review and Bayesian network meta-analysis. Evid Based Complement Alternat Med. (2021) 2021:9997516. 10.1155/2021/9997516 34221095 PMC8225439

[18] Lee EJ. The effect of auricular acupressure and positive group psychotherapy with motivational interviewing for smoking cessation in Korean adults. Holist Nurs Pract. (2020) 34:113–120. 10.1097/HNP.0000000000000348 31567305

[19] Chai XY Yang JS Liu Z Chen F Yuan GH Wu Y . Wang YY. Effect of the different smoking cessation regimens with acupuncture on smoking withdrawal and their influence factors: a multi-center randomized controlled trial. Zhongguo Zhen Jiu. (2019) 39:1255–61. 10.13703/j.0255-2930.2019.12.001 31820598

[20] MacPherson H Altman DG Hammerschlag R Youping L Taixiang W White A . Revised STandards for Reporting Interventions in Clinical Trials of Acupuncture (STRICTA): extending the CONSORT statement. J Evid Based Med. (2010) 3:140–55. 10.1111/j.1756-5391.2010.01086.x 21349059

[21] GB/T 13734-2008. Nomenclature and location of auricular points. Beijing, China: Standards Press of China. (2008).

[22] Liu Z Wang YY Wu Y Yang JS. Condition and effectiveness evaluation of acupuncture for smoking cessation. Zhongguo Zhen Jiu. (2015) 35:851–7. 10.1155/2015/189694 26571912

[23] Wang C Xiao D Wu SN Chu SL. Chinese clinical smoking cessation guidelines (2015). Chinese Journal of Health Management. (2016) 10:88–95. 10.3760/cma.j.issn.1674-0815.2016.02.00327536666

[24] Jang S Lee JA Jang BH Shin YC Ko SG Park S. Clinical effectiveness of traditional and complementary medicine interventions in combination with nicotine replacement therapy on smoking cessation: a randomized controlled pilot trial. J Altern Complement Med. (2019) 25:526–34. 10.1089/acm.2019.0009 31017453

[25] Li SJ Qu NN Wang XX Zheng X Zhao KM. Research progress on acupuncture and moxibustion for treatment of tobacco dependence. Chin Arch Tradit Chin Med. (2020) 38:93–6.

[26] Zhang FF Wang YY Liu C Wu Y Yang JS. Analysis of clinical acupoint selection rules for acupuncture for smoking cessation. Shanghai J Acupunct Moxibustion. (2016) 35:230–2. 10.13460/j.issn.1005-0957.2016.02.0230

[27] Liu Z Wang YY Wu Y Zhang F. Qi JL, Yang JS. Modern literature study on smoking cessation with acupuncture. J Trad Chin Med. (2015) 56:2116–20. 10.13288/j.11-2166/r.2015.24.012

[28] Wang YY Liu Z Wu Y Zhang O Chen M Huang LL . Acupuncture for smoking cessation in Hong Kong: a prospective multicenter observational study. Evid Based Complement Alternat Med. (2016) 2016:2865831. 10.1155/2016/2865831 28003848 PMC5149689

[29] Zhang Y Liu XJ. Clinical study of electroacupuncture at Baihui and Yintang on 34 cases of Adolescents with Internet addiction. Hunan J Trad Chin Med. (2017) 33:110–111 + 126. 10.16808/j.cnki.issn1003-7705.2017.07.049

[30] Huang CR Zhao CY. Clinical Observation on 78 Cases of Quitting Smoking by Acupuncture at Lieque, Zhaohai and Auricular Points. Inner Mongolia J Trad Chin Med. (2015) 34:102–3. 10.16040/j.cnki.cn15-1101.2015.02.105

[31] Lei ZP. A summary of 108 cases of smoking cessation with acupuncture. Guangxi J Trad Chin Med. (1986) 10:34–5.

[32] Bier ID Wilson J Studt P Shakleton M. Auricular acupuncture, education, and smoking cessation: a randomized, sham-controlled trial. Am J Public Health. (2002) 92:1642–7. 10.2105/AJPH.92.10.1642 12356614 PMC1447300

[33] Fu XQ. 150 cases of quitting smoking with ear pressure. Zhongguo Zhen Jiu. (1991) 51.

[34] Hyun S Huh H Kang NG. Effectiveness of auricular acupuncture combined with nicotine replacement therapy for smoking cessation. Tob Induc Dis. (2018) 16:40. 10.18332/tid/94328 31516439 PMC6661847

